# Crystal structure of fenclorim

**DOI:** 10.1107/S2056989015016187

**Published:** 2015-09-12

**Authors:** Eunjin Kwon, Jineun Kim, Gihaeng Kang, Tae Ho Kim

**Affiliations:** a Department of Chemistry and Research Institute of Natural Sciences, Gyeongsang National University, Jinju 660-701, Republic of Korea

**Keywords:** crystal structure, herbicide, fenclorim, pyrimidine, C—Cl⋯π inter­actions, π–π inter­actions, hydrogen bonding

## Abstract

In the title compound, C_10_H_6_Cl_2_N_2_ (systematic name: 4,6-di­chloro-2-phenyl­pyrimidine), which is used commercially as the herbicide safener, fenclorim, the dihedral angle between the di­chloro­pyrimidyl and phenyl rings is 9.45 (10)°. In the crystal, C—H⋯N hydrogen bonds link adjacent mol­ecules, forming chains along the *c-*axis direction. In addition, weak inter­molecular C—Cl⋯π [3.6185 (10) Å] and π–π [3.8796 (11) Å] inter­actions are present, forming a three-dimensional network.

## Related literature   

For information on the herbicidal properties of the title compound, see: Wu *et al.* (1999 ▸). For a related crystal structure, see: Leban & Polanc (1992 ▸).
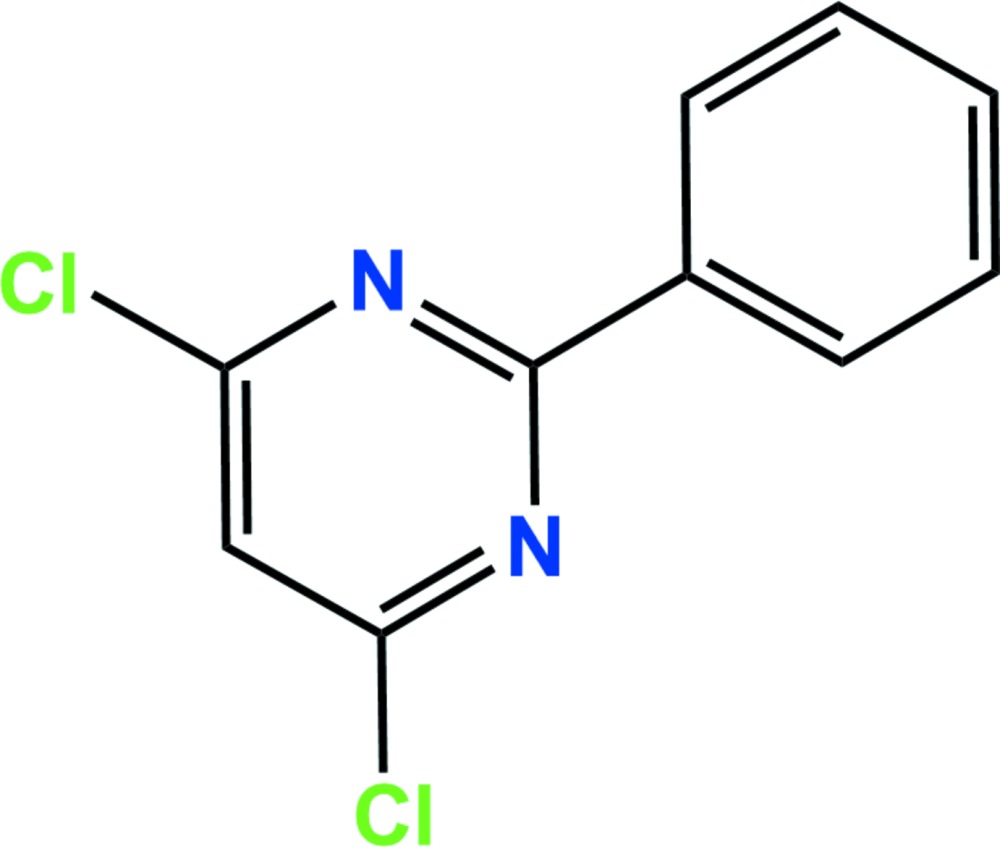


## Experimental   

### Crystal data  

C_10_H_6_Cl_2_N_2_
*M* *_r_* = 225.07
Monoclinic, 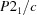

*a* = 5.6210 (6) Å
*b* = 17.0659 (18) Å
*c* = 10.2582 (12) Å
β = 99.690 (6)°
*V* = 970.00 (19) Å^3^
*Z* = 4
Mo *K*α radiation
μ = 0.62 mm^−1^
*T* = 173 K
0.16 × 0.06 × 0.04 mm

### Data collection  

Bruker APEXII CCD diffractometer
Absorption correction: multi-scan (*SADABS*; Bruker, 2013 ▸) *T*_min_ = 0.690, *T*_max_ = 0.746
9071 measured reflections
2212 independent reflections
1750 reflections with *I* > 2σ(*I*)
*R*_int_ = 0.034

### Refinement  

*R*[*F* ^2^ > 2σ(*F* ^2^)] = 0.034
*wR*(*F* ^2^) = 0.077
*S* = 1.07
2212 reflections
127 parameters
H-atom parameters constrained
Δρ_max_ = 0.28 e Å^−3^
Δρ_min_ = −0.23 e Å^−3^

### 

Data collection: *APEX2* (Bruker, 2013 ▸); cell refinement: *SAINT* (Bruker, 2013 ▸); data reduction: *SAINT*; program(s) used to solve structure: *SHELXS97* (Sheldrick, 2008 ▸); program(s) used to refine structure: *SHELXL2013* (Sheldrick, 2015 ▸); molecular graphics: *DIAMOND* (Brandenburg, 2010 ▸); software used to prepare material for publication: *SHELXTL* (Sheldrick, 2008 ▸).

## Supplementary Material

Crystal structure: contains datablock(s) global, I. DOI: 10.1107/S2056989015016187/sj5469sup1.cif

Structure factors: contains datablock(s) I. DOI: 10.1107/S2056989015016187/sj5469Isup2.hkl

Supporting information file. DOI: 10.1107/S2056989015016187/sj5469Isup3.cml

**Fig. 1.**
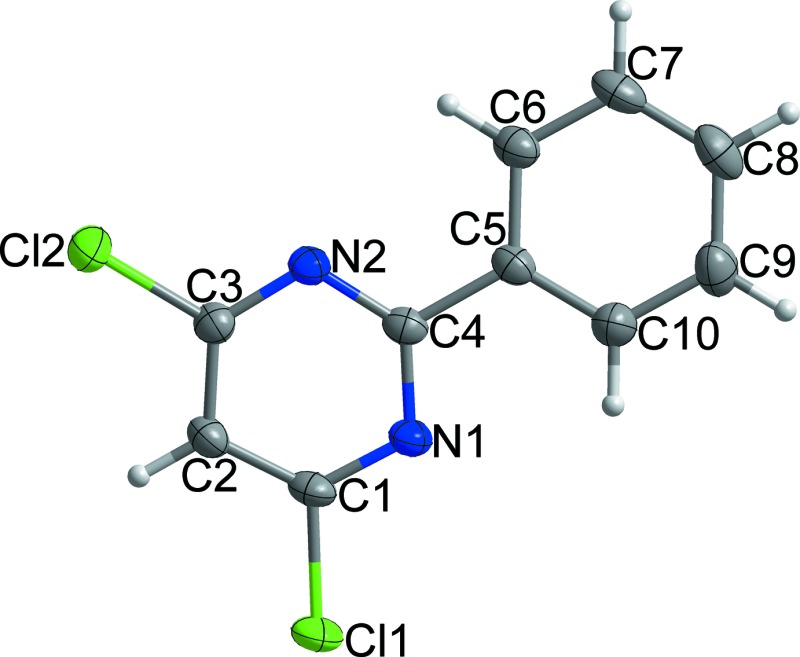
The asymmetric unit of the title compound with the atom numbering scheme. Displacement ellipsoids are drawn at the 50% probability level. H atoms are shown as small spheres of arbitrary radius.

**Fig. 2.**
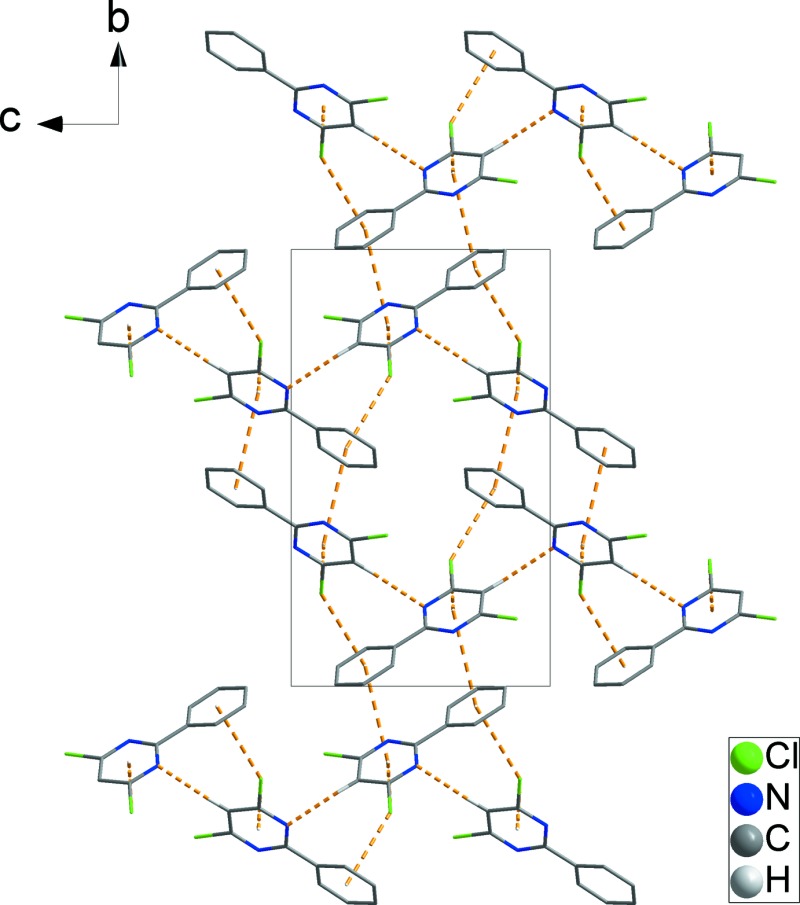
Crystal packing viewed along the a axis. The intermolecular interactions are shown as dashed lines.

. DOI: 10.1107/S2056989015016187/sj5469fig1.tif
The asymmetric unit of the title compound with the atom numbering scheme. Displacement ellipsoids are drawn at the 50% probability level. H atoms are shown as small spheres of arbitrary radius.

a . DOI: 10.1107/S2056989015016187/sj5469fig2.tif
Crystal packing viewed along the *a* axis. The inter­molecular inter­actions are shown as dashed lines.

CCDC reference: 1421258

Additional supporting information:  crystallographic information; 3D view; checkCIF report

## Figures and Tables

**Table 1:** Hydrogen-bond geometry (, )

*D*H*A*	*D*H	H*A*	*D* *A*	*D*H*A*
C2H2N2^i^	0.95	2.46	3.317(2)	151

Symmetry code: (i) 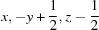.

## supplementary crystallographic information

### S1. Comment

Fenclorim [systematic name: 4,6-dichloro-2-phenylpyrimidine] is a herbicide safener that is used in many rice-producing countries to protect rice plants from damage likely to be caused by the chloroacetanilide herbicide pretilachlor. (Wu *et al.*, 1999). The dihedral angle between the dichloropyrimidyl and phenyl rings is 9.45 (10)°. All bond lengths and bond angles are normal and comparable to those observed in a similar crystal structure (Leban & Polanc, 1992).

In the crystal structure (Fig. 2), C–H···N hydrogen bonds (Table 1) link adjacent molecules, forming a one-dimensional chains along the *c*-axis. In addition, weak intermolecular C3–Cl2···*Cg*2^iii^ [Cl2···Cg2 = 3.6185 (10) Å] (*Cg2* is the centroid of the C5–C10 ring) and *Cg*1···*Cg*2^iv^ [Cg1···Cg2 = 3.8796 (11) Å] interactions are present (Cg1 is the centroid of the N1,N2,C1–C4 ring), forming a three-dimensional network [symmetry codes: (ii), *x* - 1, *y*, *z*; (iii), *x* - 1, -*y* + 1/2, *z* - 1/2; (iv), -*x* + 1, -*y* + 1, -*z*].

### S2. Experimental

The title compound was purchased from the Dr. Ehrenstorfer GmbH Company. Slow evaporation of a solution in CH_2_Cl_2_ gave single crystals suitable for X-ray analysis.

### S3. Refinement

All H-atoms were positioned geometrically and refined using a riding model with d(C—H) = 0.95 Å, *U*_iso_ = 1.2*U*_eq_(C) for the aromatic C—H.

### Crystal data

**Table:**

C_10_H_6_Cl_2_N_2_	*F*(000) = 456
*M**_r_* = 225.07	*D*_x_ = 1.541 Mg m^−^^3^
Monoclinic, *P*2_1_/*c*	Mo *K*α radiation, λ = 0.71073 Å
*a* = 5.6210 (6) Å	Cell parameters from 2286 reflections
*b* = 17.0659 (18) Å	θ = 2.3–25.9°
*c* = 10.2582 (12) Å	µ = 0.62 mm^−^^1^
β = 99.690 (6)°	*T* = 173 K
*V* = 970.00 (19) Å^3^	Plate, colourless
*Z* = 4	0.16 × 0.06 × 0.04 mm

### Data collection

**Table:**

Bruker APEXII CCD diffractometer	1750 reflections with *I* > 2σ(*I*)
φ and ω scans	*R*_int_ = 0.034
Absorption correction: multi-scan (*SADABS*; Bruker, 2013)	θ_max_ = 27.5°, θ_min_ = 2.3°
*T*_min_ = 0.690, *T*_max_ = 0.746	*h* = −6→7
9071 measured reflections	*k* = −19→22
2212 independent reflections	*l* = −13→9

### Refinement

**Table:**

Refinement on *F*^2^	0 restraints
Least-squares matrix: full	Hydrogen site location: inferred from neighbouring sites
*R*[*F*^2^ > 2σ(*F*^2^)] = 0.034	H-atom parameters constrained
*wR*(*F*^2^) = 0.077	*w* = 1/[σ^2^(*F*_o_^2^) + (0.0277*P*)^2^ + 0.269*P*] where *P* = (*F*_o_^2^ + 2*F*_c_^2^)/3
*S* = 1.07	(Δ/σ)_max_ < 0.001
2212 reflections	Δρ_max_ = 0.28 e Å^−^^3^
127 parameters	Δρ_min_ = −0.23 e Å^−^^3^

### Special details

**Table:**

Geometry. All e.s.d.'s (except the e.s.d. in the dihedral angle between two l.s. planes) are estimated using the full covariance matrix. The cell e.s.d.'s are taken into account individually in the estimation of e.s.d.'s in distances, angles and torsion angles; correlations between e.s.d.'s in cell parameters are only used when they are defined by crystal symmetry. An approximate (isotropic) treatment of cell e.s.d.'s is used for estimating e.s.d.'s involving l.s. planes.

### Fractional atomic coordinates and isotropic or equivalent isotropic displacement parameters (Å^2^)

**Table:**

	*x*	*y*	*z*	*U*_iso_*/*U*_eq_	
Cl1	0.65642 (9)	0.34546 (3)	−0.36827 (4)	0.04016 (15)	
Cl2	0.00001 (9)	0.21187 (3)	−0.11631 (4)	0.03800 (15)	
N1	0.6078 (2)	0.37521 (8)	−0.12511 (13)	0.0265 (3)	
N2	0.3094 (2)	0.31826 (8)	−0.01497 (13)	0.0251 (3)	
C1	0.5151 (3)	0.33485 (10)	−0.23126 (16)	0.0266 (4)	
C2	0.3205 (3)	0.28518 (10)	−0.24028 (16)	0.0274 (4)	
H2	0.2551	0.2580	−0.3190	0.033*	
C3	0.2296 (3)	0.27863 (10)	−0.12406 (16)	0.0258 (4)	
C4	0.4978 (3)	0.36583 (9)	−0.01979 (15)	0.0239 (4)	
C5	0.5944 (3)	0.41068 (10)	0.10104 (15)	0.0248 (4)	
C6	0.4715 (3)	0.41288 (10)	0.20807 (16)	0.0294 (4)	
H6	0.3240	0.3851	0.2044	0.035*	
C7	0.5644 (4)	0.45554 (11)	0.31979 (17)	0.0355 (4)	
H7	0.4809	0.4566	0.3929	0.043*	
C8	0.7773 (4)	0.49654 (12)	0.32573 (18)	0.0381 (5)	
H8	0.8395	0.5259	0.4026	0.046*	
C9	0.9000 (3)	0.49498 (12)	0.22019 (19)	0.0391 (5)	
H9	1.0460	0.5236	0.2239	0.047*	
C10	0.8099 (3)	0.45159 (11)	0.10867 (18)	0.0332 (4)	
H10	0.8964	0.4498	0.0367	0.040*	

### Atomic displacement parameters (Å^2^)

**Table:**

	*U*^11^	*U*^22^	*U*^33^	*U*^12^	*U*^13^	*U*^23^
Cl1	0.0519 (3)	0.0482 (3)	0.0248 (2)	−0.0100 (2)	0.0192 (2)	−0.0056 (2)
Cl2	0.0424 (3)	0.0384 (3)	0.0344 (3)	−0.0145 (2)	0.0099 (2)	−0.00277 (19)
N1	0.0314 (8)	0.0280 (8)	0.0213 (7)	−0.0007 (7)	0.0077 (6)	−0.0002 (6)
N2	0.0290 (8)	0.0250 (8)	0.0217 (7)	−0.0008 (6)	0.0056 (6)	0.0012 (6)
C1	0.0352 (10)	0.0268 (9)	0.0194 (8)	0.0042 (8)	0.0095 (7)	0.0021 (7)
C2	0.0346 (10)	0.0265 (9)	0.0210 (8)	−0.0003 (8)	0.0042 (7)	−0.0027 (7)
C3	0.0296 (9)	0.0234 (9)	0.0244 (8)	−0.0008 (7)	0.0043 (7)	0.0021 (7)
C4	0.0278 (9)	0.0240 (9)	0.0200 (8)	0.0047 (7)	0.0046 (7)	0.0033 (6)
C5	0.0307 (9)	0.0226 (9)	0.0209 (8)	0.0032 (7)	0.0035 (7)	0.0011 (6)
C6	0.0375 (10)	0.0264 (9)	0.0253 (9)	0.0013 (8)	0.0080 (7)	0.0005 (7)
C7	0.0500 (12)	0.0366 (11)	0.0206 (9)	0.0080 (9)	0.0078 (8)	−0.0013 (7)
C8	0.0442 (12)	0.0383 (11)	0.0276 (9)	0.0086 (10)	−0.0063 (8)	−0.0079 (8)
C9	0.0323 (10)	0.0415 (12)	0.0406 (11)	−0.0028 (9)	−0.0019 (8)	−0.0081 (9)
C10	0.0316 (10)	0.0375 (11)	0.0311 (9)	−0.0021 (8)	0.0073 (8)	−0.0037 (8)

### Geometric parameters (Å, º)

**Table:**

Cl1—C1	1.7362 (17)	C5—C6	1.393 (2)
Cl2—C3	1.7333 (17)	C6—C7	1.384 (2)
N1—C1	1.319 (2)	C6—H6	0.9500
N1—C4	1.342 (2)	C7—C8	1.379 (3)
N2—C3	1.320 (2)	C7—H7	0.9500
N2—C4	1.342 (2)	C8—C9	1.378 (3)
C1—C2	1.375 (2)	C8—H8	0.9500
C2—C3	1.379 (2)	C9—C10	1.385 (2)
C2—H2	0.9500	C9—H9	0.9500
C4—C5	1.480 (2)	C10—H10	0.9500
C5—C10	1.389 (2)		
			
C1—N1—C4	115.52 (14)	C6—C5—C4	120.90 (15)
C3—N2—C4	115.88 (14)	C7—C6—C5	119.93 (17)
N1—C1—C2	125.22 (15)	C7—C6—H6	120.0
N1—C1—Cl1	116.28 (13)	C5—C6—H6	120.0
C2—C1—Cl1	118.49 (13)	C8—C7—C6	120.49 (17)
C1—C2—C3	113.49 (15)	C8—C7—H7	119.8
C1—C2—H2	123.3	C6—C7—H7	119.8
C3—C2—H2	123.3	C9—C8—C7	120.06 (17)
N2—C3—C2	124.69 (16)	C9—C8—H8	120.0
N2—C3—Cl2	116.58 (13)	C7—C8—H8	120.0
C2—C3—Cl2	118.70 (13)	C8—C9—C10	119.83 (18)
N1—C4—N2	125.13 (15)	C8—C9—H9	120.1
N1—C4—C5	117.31 (15)	C10—C9—H9	120.1
N2—C4—C5	117.55 (14)	C9—C10—C5	120.63 (17)
C10—C5—C6	119.06 (16)	C9—C10—H10	119.7
C10—C5—C4	120.04 (15)	C5—C10—H10	119.7
			
C4—N1—C1—C2	−0.4 (2)	N1—C4—C5—C10	−8.8 (2)
C4—N1—C1—Cl1	−179.46 (12)	N2—C4—C5—C10	170.39 (16)
N1—C1—C2—C3	−1.8 (3)	N1—C4—C5—C6	170.99 (15)
Cl1—C1—C2—C3	177.25 (12)	N2—C4—C5—C6	−9.9 (2)
C4—N2—C3—C2	−2.3 (2)	C10—C5—C6—C7	0.1 (3)
C4—N2—C3—Cl2	175.70 (12)	C4—C5—C6—C7	−179.64 (15)
C1—C2—C3—N2	3.2 (3)	C5—C6—C7—C8	0.5 (3)
C1—C2—C3—Cl2	−174.72 (13)	C6—C7—C8—C9	−0.3 (3)
C1—N1—C4—N2	1.6 (2)	C7—C8—C9—C10	−0.6 (3)
C1—N1—C4—C5	−179.38 (14)	C8—C9—C10—C5	1.2 (3)
C3—N2—C4—N1	−0.3 (2)	C6—C5—C10—C9	−1.0 (3)
C3—N2—C4—C5	−179.36 (14)	C4—C5—C10—C9	178.79 (16)

### Hydrogen-bond geometry (Å, º)

**Table:**

*D*—H···*A*	*D*—H	H···*A*	*D*···*A*	*D*—H···*A*
C2—H2···N2^i^	0.95	2.46	3.317 (2)	151

Symmetry code: (i) *x*, −*y*+1/2, *z*−1/2.
