# Supplementary figures and images for: Crystal structure of fenclorim
Source: Acta Crystallogr E Crystallogr Commun. 2015 Sep 12;71(Pt 10):o714. doi: 10.1107/S2056989015016187 (PMC4647348; doi:10.1107/S2056989015016187)

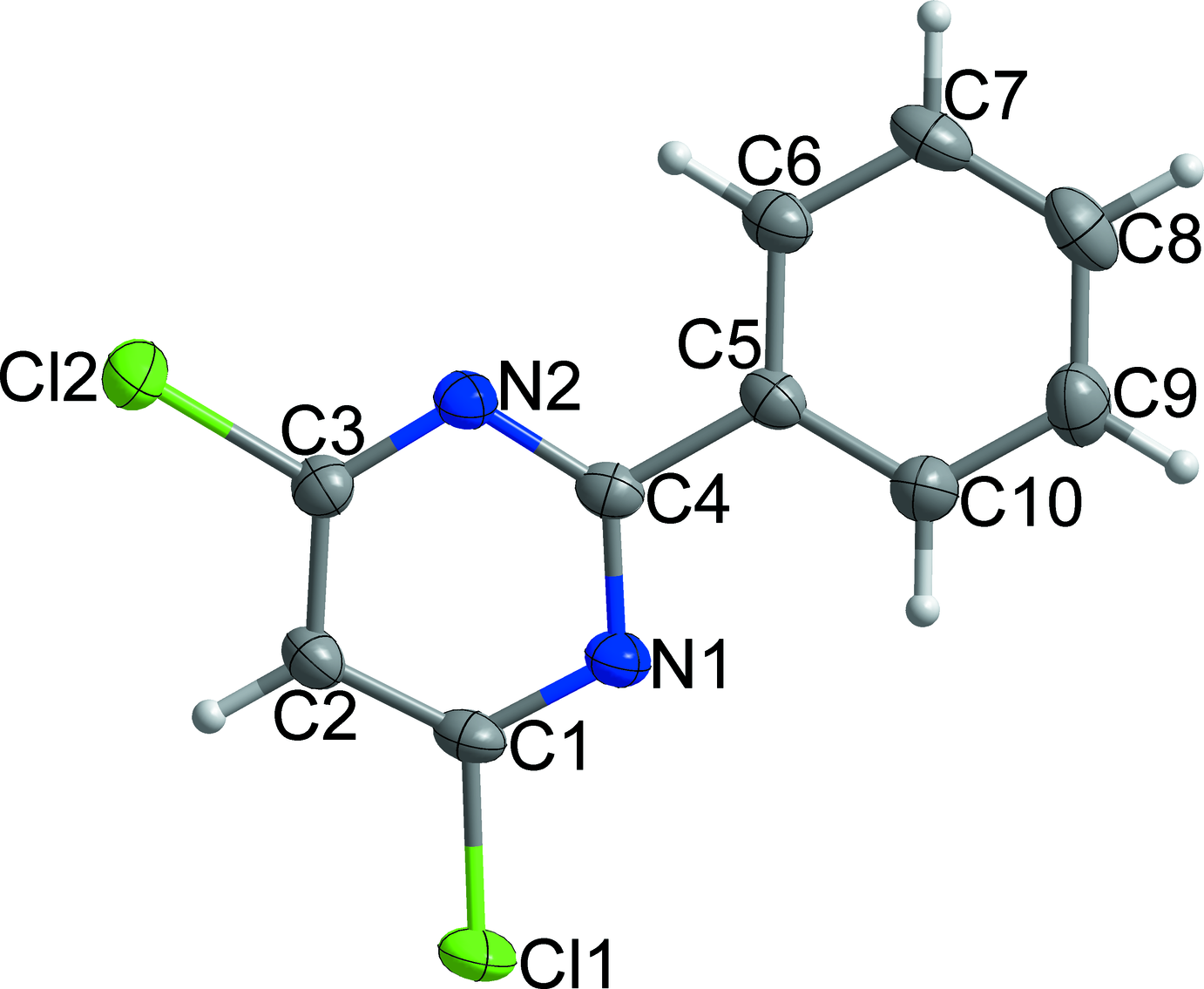

Supplement: Supplementary file 4 [file e-71-0o714-fig1.tif]

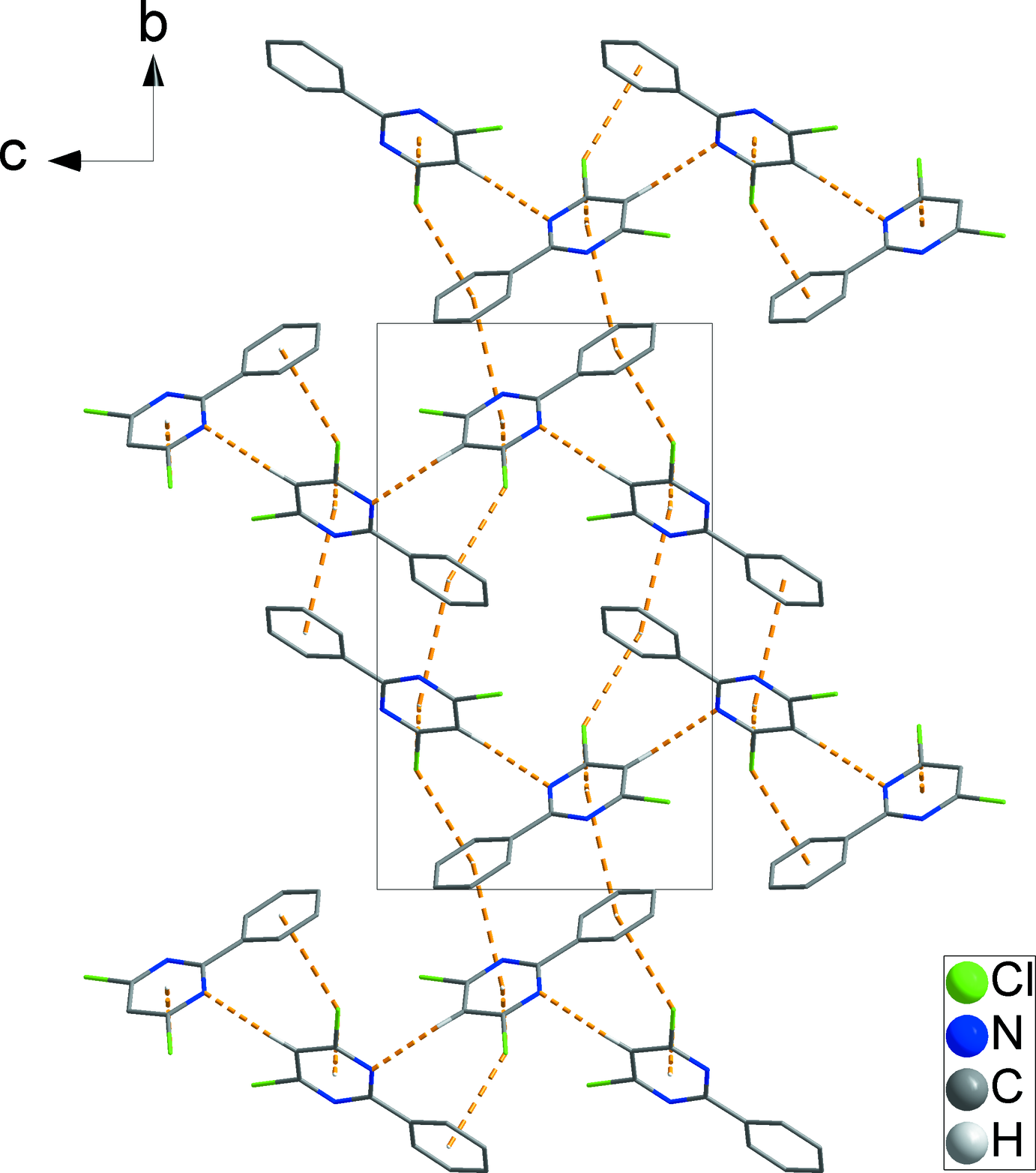

Supplement: Supplementary file 5 [file e-71-0o714-fig2.tif]
